# Developing a Functional Triticale Noodle by Incorporating Silkworm (*Antheraea pernyi* and *Bombyx mori*) Pupae

**DOI:** 10.3390/foods14132282

**Published:** 2025-06-27

**Authors:** Yu Liu, Ruixin Liu, Onanong Phuseerit, Hua Li, Sirithon Siriamornpun

**Affiliations:** 1 Department of Cuisine and Nutrition, Yangzhou University, Yangzhou 225127, China; mx120231288@stu.yzu.edu.cn (Y.L.); liurx@yzu.edu.cn (R.L.); 2 Key Laboratory of Chinese Cuisine Intangible Cultural Heritage Technology Inheritance, Ministry of Culture and Tourism, Yangzhou 225127, China; 3 Department of Science and Technology, Faculty of Liberal Arts and Science, Roi-Et Rajabhat University, Sela Phum District, Roi-Et 45120, Thailand; onanong.p@reru.ac.th; 4 Research Unit of Thai Food Innovation, Department of Food Technology and Nutrition, Mahasarakham University, Kantarawichai, Maha Sarakham 44150, Thailand

**Keywords:** silkworm pupa, edible insect, entomophagy, triticale noodle, biological activity, starch digestibility, protein digestibility

## Abstract

To enhance the nutritional and functional properties of triticale noodles, this study assessed their proximate composition, sensory attributes, and antioxidant activity, along with protein and starch digestibilities when supplemented with varying concentrations (0%, 5%, 10%, 15%) of silkworm (*Antheraea pernyi* and *Bombyx mori*) pupa powder (SP). Incorporating SP into triticale noodles led to significant enhancements in protein, fat, and ash contents (*p* < 0.05). The addition of SP may also lead to noticeable color and texture differences by decreasing the lightness (L*) and increasing hardness and springiness in noodles, as well as the flavor difference revealed by E-nose and E-tongue analysis. Furthermore, the total phenolic content and abilities to scavenge DPPH and ABTS radicals improved as the SP level increased. Compared to the control, the SP-fortified noodles had a significantly high in vitro protein digestibility but a low estimated glycemic index. Overall, due to their enhanced nutritional value and bioactivities, SP triticale noodles could be regarded as a healthier alternative to traditional noodles.

## 1. Introduction

Noodles are valued globally for their convenience and nutritional potential, serving as a staple food in many Asian countries [1]. However, refined flour products, like those from wheat, corn, and rice, can trigger a rapid rise in blood glucose levels after eating, posing health risks, particularly for individuals with diabetes. This glycemic response is largely attributable to their high content of rapidly digestible carbohydrates. Additionally, these noodles frequently lack some essential amino acids, especially lysine and methionine, crucial for holistic health [2].

As the world’s population is predicted to exceed 10 billion by 2060, the search for sustainable and nutritious food sources has become imperative [3]. Recognized by the Food and Agriculture Organization of the United Nations, the consumption of edible insects for their low environmental footprint and high nutritional value marks them as a viable solution to global food insecurity [4,5]. As by-products of the silk industry in nations including China and Thailand, silkworm pupae (SP) are prized for their significant protein content, amino acids (methionine, valine, and phenylalanine), and health-promoting components such as α-linolenic acid and α-glucosidase inhibitors [6,7].

Unfamiliarity and perceived unpalatability of insects can impede their acceptance as food [8]. Research indicated that mitigating disgust and enhancing the appeal of insects may encourage consumer trials. In the West, consumers tend to eat insects if they are in non-recognizable forms, like powders [9]. Ground SP can be utilized to enrich products such as biscuits [10,11], pasta [12,13,14], chicken meat spread [6], and breakfast cereals [15]. Previous studies have found that adding edible insect powder to products can reduce postprandial glucose response [16] and enhance indispensable amino acids [17]. Consequently, enrichment is beneficial for healthy products whose ingredients can cover up the color of insect powders effectively and can be combined with insect proteins successfully.

Triticale (*Triticosecale wittmack*), a hybrid of wheat and rye, has gained popularity in noodle production due to its rich nutrient content and bioactive compounds [18]. This cereal offers a valuable alternative to traditional grains, enhancing nutritional profiles and bioactivity in noodle products [19]. Owing to its naturally darker color and superior nutritional composition, triticale noodles are ideal for enrichment with insect powders. Consequently, this study aimed to examine how SP enrichment influences multiple quality parameters of triticale noodles, including their nutritional value, sensory performance, free radical scavenging ability, and macronutrient digestibility. The integration of triticale and SP may be helpful in developing functional foods that address both nutritional deficiencies and sustainability issues.

## 2. Materials and Methods

### 2.1. Materials and Chemicals

Triticale (variety: ND2201) was sourced from Huinong Fumin Technology (Beijing, China). Black *Antheraea pernyi* pupae (BAP) and yellow *Antheraea pernyi* pupae (YAP) were acquired from a sericulture farm (Liaoning, China), and *Bombyx mori* pupae (BP) was obtained from a breeding base (Shandong, China). DPPH, ABTS, gallic acid, pancreatin, pepsin, and porcine bile salt were obtained from Yuanye Biotechnology (Shanghai, China). Amyloglucosidase and α-amylase were purchased from Aladdin Biochemical Technology (Shanghai, China). All additional chemicals of analytical grade were purchased from Sinopharm Chemical Reagent (Shanghai, China).

### 2.2. Preparation of Triticale Flour and Insect Powders

**Figure 1:**
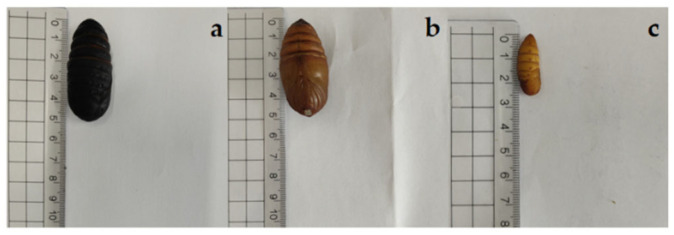
Insects used for triticale noodle production. (**a**) Black *Antheraea pernyi* pupae; (**b**) yellow *Antheraea pernyi* pupae; (**c**) *Bombyx mori* pupae.

The triticale grains were milled and sieved through an 80-mesh screen. The resulting triticale flour (TF) was stored at ambient temperature until use, while for the preparation of insect powders, the pupae (see Figure 1) were lyophilized (−50 °C, 48 h) in a dryer (LYOQUEST-55, Azibil Telstar, Shanghai, China) and then ground. After passing through an 80-mesh sieve, the dried pupa powders were stored at −18 °C until use.

### 2.3. Preparation of Triticale Noodles Fortified with Silkworm Pupae

The triticale noodles supplemented with various SP were prepared as follows: TF was well blended for 3 min with SP in various proportions (5%, 10%, and 15%) as indicated in Table 1, and then water (40% of the TF weight) was added to make a dough. The dough moisture content was determined to be 30.85 ± 1.79% using an oven-drying method. After resting for 15 min, the dough was pressed and cut using a home noodle-making machine (DM-YMJ00, Junxifu Company, Jinhua, Zhejiang, China). Similarly, the control sample (C)—that is, the triticale noodles without adding insect powder—was prepared following the aforementioned procedure. After cooking the noodles in boiling water for 3 min, one part of the samples was immediately analyzed for color, texture properties, and E-nose and E-tongue data. Another part was freeze-dried and ground. The ground noodles were passed through an 80-mesh sieve, sealed, and preserved in a desiccator at room temperature until used.

### 2.4. Proximate Analysis

Nutritional components (protein, fat, and ash) in the cooked noodles were measured in accordance with AOAC methods [20], and carbohydrate content was estimated by the difference method.

### 2.5. Texture Profile Analysis

Texture properties of the cooked noodles were assessed using Wang et al.’s protocol [21] using a TA.XT plus texture analyzer (SMS, Godalming, UK) configured with a P/36R probe. The noodles were cooked for 3 min in boiled water, and five strips of the cooked noodles were placed parallel on a metal platform. Testing conditions included pre-test (2.0 mm/s), test (0.8 mm/s), and post-test (0.8 mm/s) speeds, 5.0 g trigger force, and 70% deformation. Each test was repeated six times.

### 2.6. Color Measurement

The noodle color was quantified using a Konica Minolta CR-400 colorimeter (Tokyo, Japan). After standard calibration, five cooked noodle strands were arranged in a linear configuration, and six replicate measurements of L* (lightness/darkness), a* (redness/greenness), and b* (yellowness/blueness) values were obtained from six distinct locations across the aligned strands for each sample.

### 2.7. Analysis of Total Phenolic Content and Antioxidant Power

#### 2.7.1. Extraction of Phenolic Compounds

Polyphenol compounds were extracted from triticale noodles using a method described by Yu et al. [22] with minor modification. Initially, 500 mg of the sample powder was extracted with 10 mL of 70% ethanol at 40 °C for 15 min under 240 W using an ultrasonic cleaner (AK-040SD, Yujie Cleaning Equipment, Shenzhen, China). After sonication, the mixture was shaken (SHZ-82A, Shengwei Experimental Instrument Factory, Changzhou, China) at 160 rpm and 40 °C for 1 h. Subsequently, the suspension was centrifuged at 6000 rpm for 25 min. The residue was extracted using the aforementioned method twice, and the collected supernatants were pooled and diluted to 25 mL using 70% ethanol. The resulting extracts were subsequently stored at −20 °C until use.

#### 2.7.2. Analysis of Total Phenolic Content

Total phenolic content (TPC) quantification was performed according to Penalver et al. [23]. In brief, 1 mL of the extract was thoroughly vortexed with 4.5 mL of 10% (*v*/*v*) Folin-Ciocalteu’s phenol reagent. After a reaction for 10 min, 5 mL of Na_2_CO_3_ solution (7.5%, *v*/*v*) was added. After 1 h of dark incubation at room temperature, reaction mixtures were spectrophotometrically analyzed at 765 nm (V-1100D, Mapada, Shanghai, China), with TPC expressed as milligram gallic acid equivalents per gram sample (mg GAE/g).

#### 2.7.3. Analysis of DPPH Radical Scavenging Activity

The DPPH value was determined following Farzana et al. [24] with slight modifications. Briefly, the extract (0.5 mL) was thoroughly vortexed with 0.1 mmol/L DPPH solution (3 mL), incubated in darkness (25 °C, 30 min), and measured at 517 nm. Results were calculated as micromolar Trolox equivalents per gram sample (μmol TE/g).

#### 2.7.4. Analysis of ABTS Radical Scavenging Activity

According to the method of Tang et al. [25], the ABTS value of the samples was assessed. Initially, the stock solution was produced by reacting equal volumes of 7 mM ABTS and 2.45 mM potassium persulfate under dark conditions for 12–16 h. Subsequently, the solution was diluted with 10 mM PBS to achieve an absorbance of 0.70 ± 0.02 at 734 nm. In the assay, the extract (100 μL) was introduced into test tubes containing the ABTS radical solution (3.9 mL). After 10 min of dark incubation, absorbance measurements were taken at 734 nm, with antioxidant capacity calculated as micromolar Trolox equivalents per gram sample (μmol TE/g).

### 2.8. Starch Digestibility Analysis

The in vitro digestion of starch was conducted using the method of Englyst et al. [26] with some alterations. The dried noodle powder (200 mg) was well mixed with sodium acetate buffer (15 mL, 0.2 M, pH 5.2). Then, amyloglucosidase (15 U/mL) and α-amylase (290 U/mL) were added to the mixtures to initiate the enzymatic hydrolysis in a water bath of 37 °C at 160 rpm. During the incubation, the supernatant (1 mL) was taken at 0, 20, 30, 60, 90, 120, 150, and 180 min, immediately mixed with 66% ethanol (2.5 mL) to terminate enzymatic activity, and centrifuged (6000 rpm, 15 min). The glucose content was determined using the DNS assay. The hydrolysis index (HI) derived from the hydrolysis curve area (white bread reference) and the estimated glycemic index (eGI) was calculated using the following equation:

eGI = 8.198 + 0.862 × HI(1)

### 2.9. Analysis of Protein Digestibility

Following Minekus et al.’s [27] formulations, simulated salivary (SSF), gastric (SGF), and intestinal (SIF) fluids were prepared for INFOGEST-compliant gastrointestinal digestion of triticale noodles. For the oral phase, 1 g of the cooked noodles was mixed with 5 mL of SSF and 250 µL of α-amylase (1500 U/mL), then subjected to 2 min incubation in a 37 °C shaking water bath (120 rpm). For the gastric phase, the mixture was diluted with SGF (1:1, *v*/*v*), supplemented with pepsin (2000 U/mL), and maintained at 37 °C (pH 3.0) for 2 h. For the intestinal phase, the mixture was diluted with SIF (1:1, *v*/*v*) supplemented with 10 mM bile salts and pancreatin (100 U/mL trypsin activity in the final mixture), followed by incubation at 37 °C and pH 7.0 for 2 h. After centrifugation (9000 rpm, 20 min) of the enzymatic hydrolysate, the precipitate was preserved at −20 °C until use. The protein digestibility (%) was quantified as the percentage ratio of soluble protein in the digests to total protein in the original sample.

### 2.10. E-Nose Analysis

The volatile substances of cooked triticale noodle samples were detected by an E-nose (PEN3, Airsense Analytics Inc., Schwerin, Germany), according to Fan’s method with a little modification [28]. The response characteristics of 10 metal sensors are shown in Table 2. The cooked sample was placed in a headspace bottle (40 mL) and equilibrated at 60 °C for 30 min to reach a steady state. The flow rate of both the chamber and injection was 400 mL/min, and the sample interval, flush, zeroing, presampling, and measurement times were 1, 60, 10, 5, and 60 s, respectively.

### 2.11. E-Tongue Analysis

Taste profile evaluation was performed using an SA402B E-tongue (Intelligent Sensor Technology, Atsugi, Japan) following an adapted methodology [29]. Taste sensors included eight gustatory indicators: primary tastes (sourness, bitterness, umami, and saltiness), mouthfeel (astringency and richness), and aftertaste components (aftertaste astringency, aftertaste-A; aftertaste bitterness, aftertaste-B). The cooked samples (5 g) were added to distilled water (30 mL), then centrifuged at 4 °C and 6000 rpm for 25 min. Beakers containing 40 mL of each supernatant were positioned on the instrument for measurement.

### 2.12. Statistical Analysis

All analyses were carried out in triplicate unless otherwise stated. Data were presented as mean ± standard deviation. Significant differences (*p* < 0.05) were determined by one-way ANOVA and the LSD multiple comparison procedure using SPSS 17.0 (IBM, Chicago, IL, USA), with graphical representations generated in Origin 2021 (OriginLab, Northampton, MA, USA). Principal component analysis (PCA) was used for dimensionality reduction of E-nose and E-tongue data. Eigenvalues, accumulative variance contributions, and component score coefficient matrix were determined, and a loading and score plot was created using Origin 2021.

## 3. Results and Discussion

### 3.1. Proximate Composition of Triticale and Silkworm Pupae

Table 3 illustrates the proximate compositions (carbohydrate, protein, fat, and ash) of the raw materials for the triticale noodle-making, i.e., TF, BAP powder, YAP powder, and BP powder. Statistical analysis confirmed significant differences in nutrient contents among these samples (*p* < 0.05). The most abundant component in TF was carbohydrate, accounting for almost 80% of dry matter. Meanwhile, the protein content of TF was 19.32%, a little higher than the value (12.30%) reported by Piazza et al. [30], which may be owing to the difference in genotypes. Distinct from TF, all the SP were rich in protein and fat, aligning with the results of Li et al. [31], which noted that proteins and fats are the predominant components in insects. Specifically, YAP powder displayed the highest protein content (57.31%), while BP powder was distinguished by its prominent fat content (32.80%). Additionally, research by Yeruva et al. [32] on BP also indicated high levels of protein (51–55%) and fat (25–32%), consistent with the results of our BP sample. Similarly, the protein and fat contents of BAP and YAP coincided with the results of Wang et al. [33]. Although SP is high in lipids, these belong to essential or functional fatty acids that provide health benefits. Several studies [34,35,36] have shown that both *B. mori* pupae and *A. pernyi* pupae contain abundant α-linolenic, linoleic, and oleic acid. Furthermore, carbohydrate levels in insect powders ranged from 11.89% to 16.38%, indicating a significant reduction compared to traditional grain flours. It is crucial to emphasize that the composition can vary depending on the species [37], their developmental stages [38], as well as their diet [34,36], and measurement techniques [39].

### 3.2. Proximate Composition of Triticale Noodles

The proximate composition of triticale noodles enriched with each SP is detailed in Table 4. As expected, with all levels of BAP, YAP, and BP in the triticale noodles, the contents of protein, fat, and ash gradually increased (*p* < 0.05), which is attributed to the higher values of these nutrients in the SP powders compared to the triticale flour. Ho et al. [40] indicated that the protein content increased when wheat flour was substituted with 7% cricket powder. Akande et al. [10] also reported that after adding two types of insects (silkworm pupa and locust), the protein content of the enriched biscuits increased 20% compared with the control. Conversely, the carbohydrate content of the noodles declined as the proportion of SP increased, a result of reducing the amount of TF used. Additionally, ash content displayed a marked increase across all samples, indicating an enhanced mineral content as well as potential contribution from chitin.

### 3.3. Texture Profile

The texture characteristics of noodles are a key factor in influencing consumers’ acceptability of the product [41]. Table 5 shows the texture profiles of the triticale noodles, including springiness, cohesiveness, hardness, chewiness, and resilience. The triticale noodles enriched with SP were characterized by a noticeably harder texture, thus requiring greater chewing force compared to the control. These findings are consistent with the result of Pasini et al. [42], who reported that the addition of 14% cricket protein extracts strengthened the hardness of durum wheat pasta, which may be a consequence of the increased protein content. The protein additives commonly used in noodle production, such as egg and broad bean protein, have been reported to enhance hardness [41], thereby corroborating the findings of this study. Another possibility was that starch and polyphenols may combine to form a complex, compressing the starch gel structure and preventing moisture absorption. High-quality Chinese noodles are preferred for their firm, elastic, and chewy texture [43]. These findings supported that the insect powders may be suitable for fortification into noodles.

Remarkably, the addition of SP was observed to have a minimal impact on the cohesiveness and resilience of the samples, with only minor differences noted. The presence of polyphenols, which form intermolecular hydrogen bonds with starch molecules, likely explains why cohesiveness and resilience remained unchanged. Furthermore, the inclusion of three different species of SP led to increased springiness values compared to the control noodles, potentially due to reduced amylose leaching, which facilitates complex formation [44]. The enhancements in various textural parameters may be correlated with the formation of new structures within the noodle matrix, arising from interactions between protein and starch.

### 3.4. Color of Triticale Noodles

**Figure 2:**
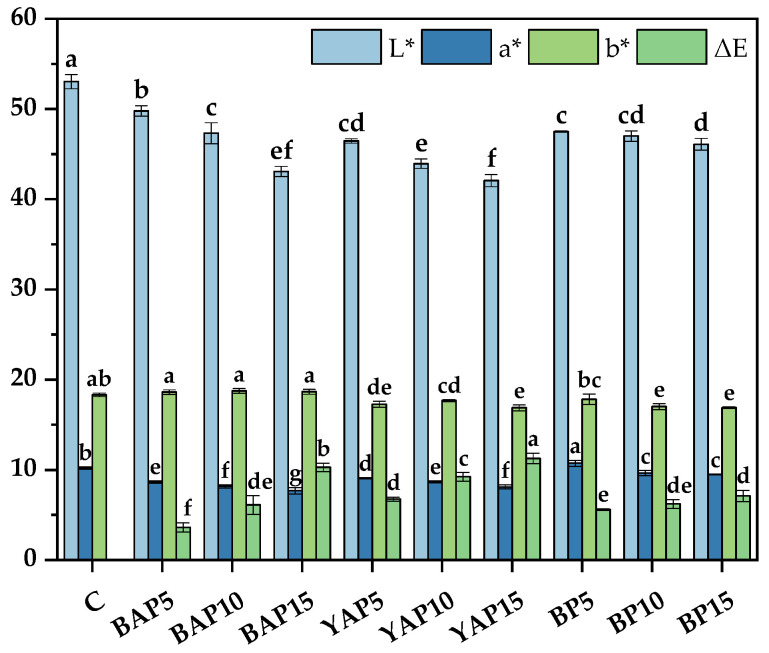
Color parameters of triticale noodles enriched with silkworm pupa powder. C represents the noodles with no supplementation of insects. BAP5, BAP10, and BAP15 represent the noodles supplemented with 5%, 10%, and 15% of black *Antheraea pernyi* pupae, respectively; YAP5, YAP10, and YAP15 represent the noodles supplemented with 5%, 10%, and 15% of yellow *Antheraea pernyi* pupae, respectively; BP5, BP10, and BP15 represent the noodles supplemented with 5%, 10%, and 15% of *Bombyx mori* pupae, respectively. Column and error bars represent the mean and standard deviation (*n* = 6), respectively. Values with different lowercases are considered significant differences (*p* < 0.05).

**Figure 3:**
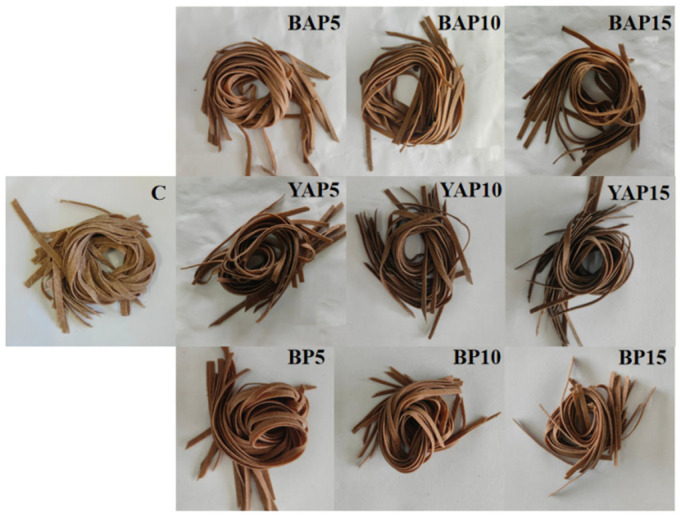
Triticale noodles with different levels (0, 5, 10, and 15%) of black *Antheraea pernyi* pupae (BAP), yellow *Antheraea pernyi* pupae (YAP), and *Bombyx mori* pupae (BP) powders. C represents the noodles with no supplementation of insects.

As well as texture profile, color is also an important physical property for consumers to consider when choosing noodles [45]. As shown in Figure 2, significant variations in total color differences (∆E), ranging from 3.62 to 11.27 (*p* < 0.05), indicated a notable change (∆E > 3.5) of color after the addition of SP. Moreover, the addition of SP led to a noticeable darkening of the triticale noodles. Particularly, triticale noodles formulated with YAP displayed progressively deeper shades as the concentration of the powder increased from 5% to 15%, as shown in Figure 3. This deepening of color may primarily result from the presence of polyphenolic pigments in the pupae. Similar observations have been noted by other authors in products enriched with insect powders [4,46]. Interestingly, despite a general trend toward decreased yellowness (b* values), triticale noodles with BAP displayed no significant difference in b* values (*p* > 0.05), directly attributable to the natural color of BAP. A marked reduction was observed in the red saturation parameter (a*) among all samples. Kowalski et al. [47] also observed a reduction in the levels of red and yellow color components in sponge cakes containing 15% and 30% buffalo worm powders. Their color resembled that of commercially available triticale noodles, which are commonly perceived as a healthier option. Thus, the darker coloring ascribed to SP may further enhance the perception of insect-based noodles as a healthy alternative among consumers.

### 3.5. Antioxidant Properties of Triticale Noodles

**Figure 4:**
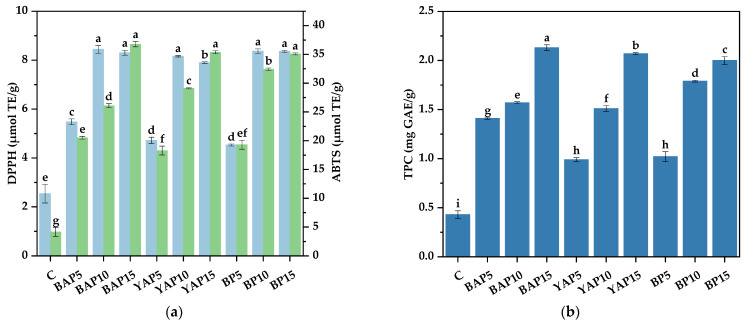
Antioxidant properties of triticale noodles enriched with silkworm pupa powder. (**a**) DPPH radical scavenging activity and ABTS radical scavenging activity; (**b**) total phenolic content (TPC). C represents the noodles with no supplementation of insects. BAP5, BAP10, and BAP15 represent the noodles supplemented with 5%, 10%, and 15% of black *Antheraea pernyi* pupae, respectively; YAP5, YAP10, and YAP15 represent the noodles supplemented with 5%, 10%, and 15% of yellow *Antheraea pernyi* pupae, respectively; BP5, BP10, and BP15 represent the noodles supplemented with 5%, 10%, and 15% of *Bombyx mori* pupae, respectively. Column and error bars represent the mean and standard deviation (*n* = 3), respectively. Values with different lowercases are considered significant differences (*p* < 0.05).

The inclusion of antioxidants in our diet is crucial for preventing the diseases related to oxidative stress, often referred to as civilization diseases, such as hypertension, atherosclerosis, and diabetes [48]. Due to the abundance of polyphenols in insects, we evaluated the antioxidant properties of the triticale noodles with SP. As shown in Figure 4, the DPPH and ABTS radical scavenging ability and TPC appreciably improved after the addition of SP. For instance, the highest TPC was found in BAP15, nearly 5.0 times higher than that in the control noodle; meanwhile, BAP15 also possessed the strongest capacities to scavenge DPPH and ABTS radicals, which were 3.3 and 8.9 times higher than the control sample, respectively (*p* < 0.05). Generally, as the level of the SP increased, the TPC content and radical scavenging abilities of the noodles became higher. The findings are not surprising, given that edible insects have high total phenolic content [49,50] and strong antioxidant abilities [51]. Similarly, sponge cakes containing 15% and 30% edible insect powder had higher polyphenol content than the standard cake [47]. Increased phenolic concentrations in insect-based triticale noodles could be due to bioactive components naturally present in SP, such as tocopherols [32] and flavonoids [52]. SP also represents a promising source of bioactive proteins and peptides [53]. These proteins and peptides are implicated in the antioxidant properties of insects, as they may modulate DPPH and ABTS free radical scavenging activities. Such variations are likely contingent upon the molecular weight of proteins or peptides and their amino acid composition [4]. Additionally, consistent with our results, muffins [46] and nut bars [50] supplemented with edible insects showed a higher antioxidant potential than the control.

### 3.6. Starch Digestibility

**Figure 5:**
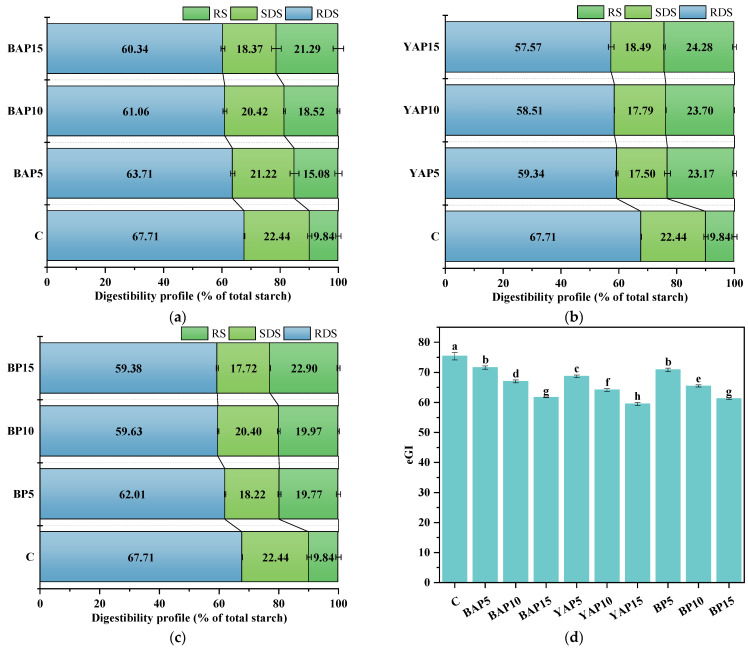
Starch digestibility of triticale noodles enriched with silkworm pupa powder. (**a**) Starch composition of BAP-noodle; (**b**) starch composition of YAP-noodle; (**c**) starch composition of BP-noodle; (**d**) eGI of silkworm pupa-noodle. C represents the noodles with no supplementation of insects. BAP5, BAP10, and BAP15 represent the noodles supplemented with 5%, 10%, and 15% of black *Antheraea pernyi* pupae, respectively; YAP5, YAP10, and YAP15 represent the noodles supplemented with 5%, 10%, and 15% of yellow *Antheraea pernyi* pupae, respectively; BP5, BP10, and BP15 represent the noodles supplemented with 5%, 10%, and 15% of *Bombyx mori* pupae, respectively. Column and error bars represent the mean and standard deviation (*n* = 3), respectively. Values with different lowercases are considered significant differences (*p* < 0.05).

Figure 5 presents the starch composition and eGI of the cooked triticale noodles. The potential impact of triticale noodles on blood sugar levels appeared substantial, owing to the RDS content being significantly higher than the SDS content. Generally, the incorporation of insect powder into triticale noodles promoted a reduction in RDS and a rise in RS content (*p* < 0.05). Moreover, a dose-dependent effect was found; that is, with the ratios of insect powders in the noodles increasing, the RS content gradually increased while the RDS content declined. Additionally, the starch digestibility of the noodles also varied with the type of SP. The highest RS and lowest RDS contents were both found in the noodles with the addition of YAP, followed by those with BP and BAP, irrespective of insect content. Studies have indicated that the interactions between starch and non-starch compounds, such as proteins and lipids, are crucial in the formation of RS, noted for its health benefits [54]. The changes in RS contents and eGI after adding SP may be partly attributed to protein–starch interactions. Specifically, the insect-derived proteins may form a physical barrier around starch granules, retarding enzymatic hydrolysis and promoting resistance starch formation, thereby reducing the eGI of triticale noodles. Additionally, the addition of SP led to an increase in the contents of fat and polyphenols, which may affect the starch digestibility and eGI.

These results highlight the important low-glycemic properties of insect powders, which are partially corroborated by the literature, with Suk et al. [16] noting that wheat flour noodles supplemented with silkworm powder were beneficial for reducing postprandial glucose response and potentially served as a carbohydrate staple for blood sugar control. Modulation of the postprandial blood glucose response via low-GI foods is associated with positive health effects, underscoring the need for further research in this domain.

### 3.7. In Vitro Protein Digestibility

**Figure 6:**
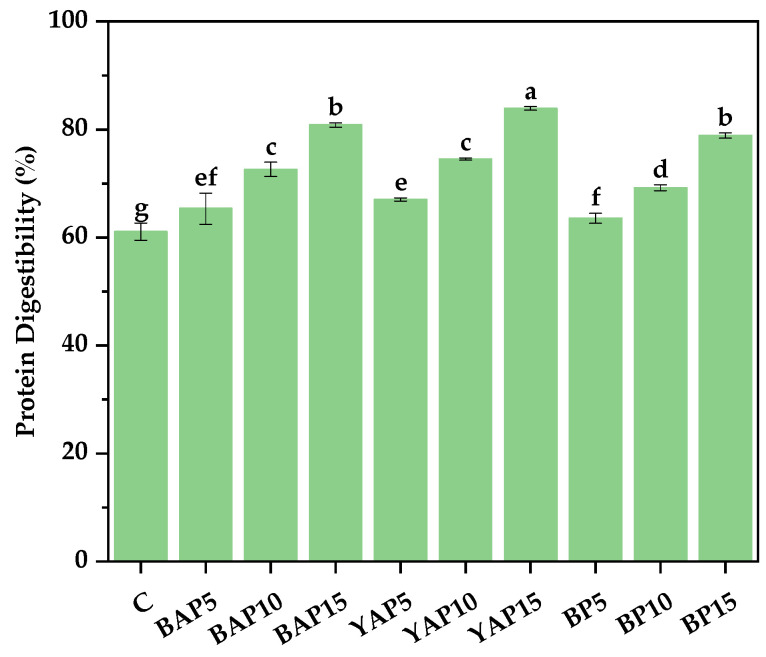
Protein digestibility of triticale noodles enriched with silkworm pupa powder. C represents the noodles with no supplementation of insects. BAP5, BAP10, and BAP15 represent the noodles supplemented with 5%, 10%, and 15% of black *Antheraea pernyi* pupae, respectively; YAP5, YAP10, and YAP15 represent the noodles supplemented with 5%, 10%, and 15% of yellow *Antheraea pernyi* pupae, respectively; BP5, BP10, and BP15 represent the noodles supplemented with 5%, 10%, and 15% of *Bombyx mori* pupae, respectively. Column and error bars represent the mean and standard deviation (*n* = 3), respectively. Values with different lowercases are considered significant differences (*p* < 0.05).

The protein digestibility of triticale noodles is illustrated in Figure 6. The addition of SP caused a significant improvement in the protein digestibility, while the triticale noodles containing BP showed lower protein digestibility than those enriched with BAP and YAP. The highest value of protein digestibility was found in the YAP15, which was up to 83.89%, 1.37-fold higher than the unsupplemented control. Mihaly Cozmuta et al. [38] reported that 15% yellow mealworm powder supplementation in biscuits enhanced protein digestibility by diluting the gluten network, yielding a less dense structure that facilitated digestive juice and protease access to the digesta. Bas et al. [55] and Ronoh et al. [56] also observed that biscuits enriched with insect powders had higher protein digestibility, which may be attributed to the high digestibility of insect proteins. The difference in protein digestibility among various foods is usually related to their protein composition and the presence of antinutrients.

### 3.8. E-Nose Analysis

**Figure 7:**
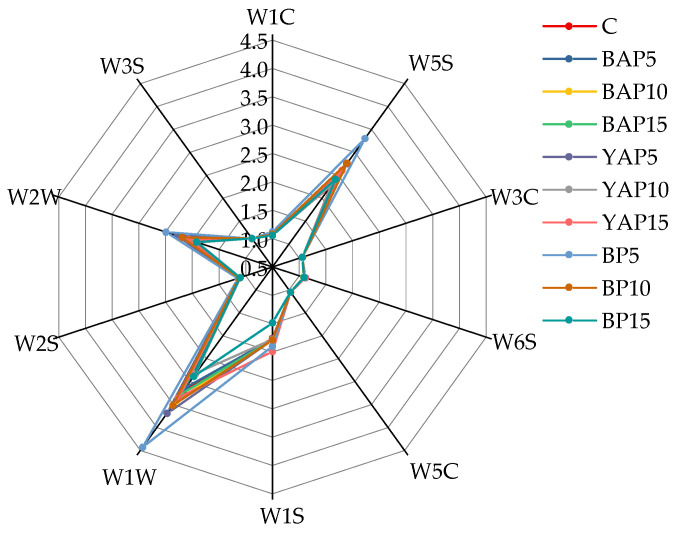
Radar chart of E-nose sensor response signal values for triticale noodles. C represents the noodles with no supplementation of insects. BAP5, BAP10, and BAP15 represent the noodles supplemented with 5%, 10%, and 15% of black *Antheraea pernyi* pupae, respectively; YAP5, YAP10, and YAP15 represent the noodles supplemented with 5%, 10%, and 15% of yellow *Antheraea pernyi* pupae, respectively; BP5, BP10, and BP15 represent the noodles supplemented with 5%, 10%, and 15% of *Bombyx mori* pupae, respectively.

The E-nose has been demonstrated to effectively detect and thoroughly analyze volatile compounds within samples [57]. The radar diagram was generated utilizing the response values of 10 metal detectors when exposed to various noodle samples. As displayed in Figure 7, the plot profiles exhibited similarity, while variations in some response values were observed, suggesting that the categories of volatile compounds were generally similar, but their contents varied. Moreover, the response values of four sensors (W5S, W1S, W1W, and W2W) were higher than those of other sensors—nitrogen oxides, methyl compounds, inorganic sulfides, terpenes, and aromatic compounds (organic sulfides)— which implied that these volatile compounds were potential contributors to the overall flavor of the triticale noodles. The radar analysis could not intuitively reflect the flavor differences of samples made from different SP, so further analysis of the data was needed.

**Figure 8:**
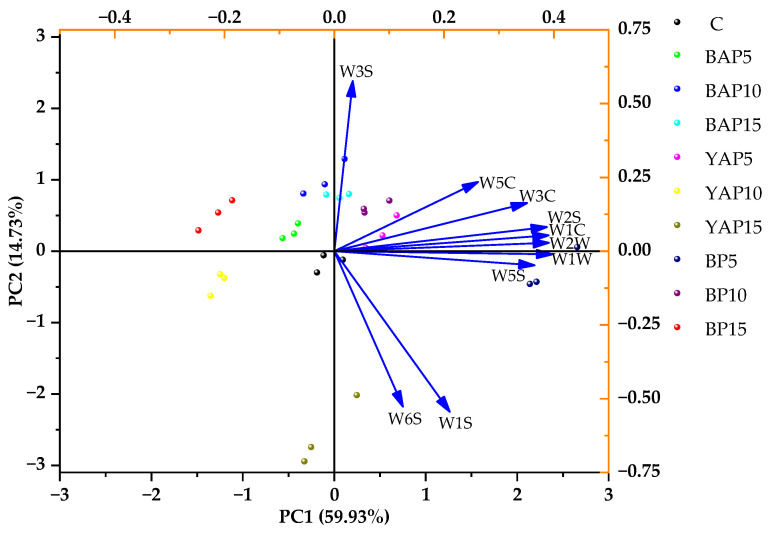
Principal component analysis score and loading plot of the E-nose for different triticale noodles. C represents the noodles with no supplementation of insects. BAP5, BAP10, and BAP15 represent the noodles supplemented with 5%, 10%, and 15% of black *Antheraea pernyi* pupae, respectively; YAP5, YAP10, and YAP15 represent the noodles supplemented with 5%, 10%, and 15% of yellow *Antheraea pernyi* pupae, respectively; BP5, BP10, and BP15 represent the noodles supplemented with 5%, 10%, and 15% of *Bombyx mori* pupae, respectively.

PCA was employed for dimensionality reduction of E-nose sensor response data, enabling effective discrimination among sample groups [58]. As shown in Figure 8, the combined variance explained by PC1 (59.93%) and PC2 (14.73%) was 74.66%, demonstrating the accuracy of the PCA results in explaining the odor data across all samples. PC1 exhibited positive correlations with all sensor responses, and the longer arrows of W1W, W1C, W2W, W2S, W5S, and W3C suggested that these odor attributes made a higher contribution to PC1, while PC2 showed a strong positive correlation with W3S and negative correlations with W6S and W1S. For each type of SP, the differences in sample point positions reflected the variations in volatile compound characteristics in the noodles under different SP concentrations. Moreover, BP5 showed evident odor differences compared to other samples. The position of BP5 was close to the arrows of W1W, W1C, W2W, W2S, and W5S, indicating higher levels of aromatic components, sulfides, terpenes, alcohols, aldehydes, ketones, and nitrogen oxides. Accordingly, E-nose technology combined with PCA may be helpful to differentiate SP-enriched noodles based on the volatile substances.

### 3.9. E-Tongue Analysis

**Figure 9:**
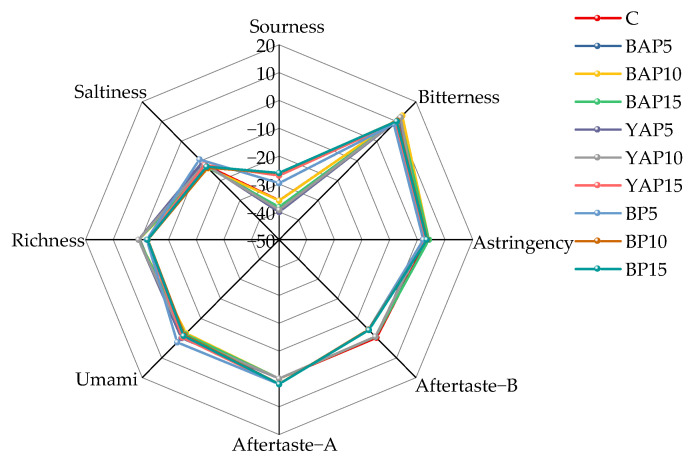
Radar chart of E-tongue sensor response signal values for triticale noodles. C represents the noodles with no supplementation of insects. BAP5, BAP10, and BAP15 represent the noodles supplemented with 5%, 10%, and 15% of black *Antheraea pernyi* pupae, respectively; YAP5, YAP10, and YAP15 represent the noodles supplemented with 5%, 10%, and 15% of yellow *Antheraea pernyi* pupae, respectively; BP5, BP10, and BP15 represent the noodles supplemented with 5%, 10%, and 15% of *Bombyx mori* pupae, respectively.

As shown in Figure 9, E-tongue analysis revealed relatively higher bitterness and astringency across all samples compared to other sensors, which may be attributed to the phenolic compounds in triticale noodles and alkaloids in SP [59]. No differences emerged with 5–15% substitutions, as triticale’s dominant bitter/astringent signals overshadowed minor variations from ingredients. The noodle matrix likely diluted substitution-derived compounds, while the E-tongue’s broad specificity hindered detection of phenolic differences. The E-tongue sensor response profiles exhibited analogous trends across samples. Visual inspection of response curves and radar charts proved insufficient for discriminating triticale noodles, necessitating multivariate statistical processing of the E-tongue data.

**Figure 10:**
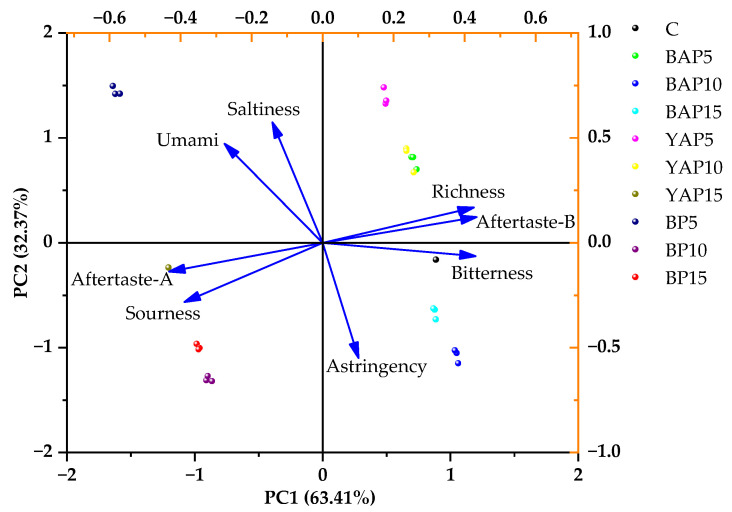
Principal component analysis score and loading plot of the E-tongue for different triticale noodles. C represents the noodles with no supplementation of insects. BAP5, BAP10, and BAP15 represent the noodles supplemented with 5%, 10%, and 15% of black *Antheraea pernyi* pupae, respectively; YAP5, YAP10, and YAP15 represent the noodles supplemented with 5%, 10%, and 15% of yellow *Antheraea pernyi* pupae, respectively; BP5, BP10, and BP15 represent the noodles supplemented with 5%, 10%, and 15% of *Bombyx mori* pupae, respectively.

As displayed in Figure 10, the first two principal components (PC1 = 63.41%, PC2 = 32.37%) collectively accounted for 95.78% of total variance, indicating that they sufficiently captured the majority of sample information. PC1 was mainly explained by sourness, bitterness, richness, aftertaste-A, and aftertaste-B, whereas PC2 showed stronger correlations to saltiness, astringency, and umami. Ten noodle samples were distributed in four different quadrants; i.e., first quadrant (YAP5, YAP10, and BAP5), second quadrant (BP5), third quadrant (YAP15, BP10, and BP15), and fourth quadrant (C, BAP10, and BAP15). With the exception of YAP10 and BAP5, the remaining samples demonstrated clear differentiation. For example, the control exhibited stronger bitterness characteristics, while BP5 displayed more pronounced saltiness and umami attributes. The observed variations may be attributed to differences in SP varieties, leading to the different chemical ingredients and tastes of triticale noodles. Generally, the PCA results validate the E-tongue as a reliable tool for triticale noodle differentiation.

## 4. Conclusions

To maximize the economic value of SP as well as strengthen the nutritional quality and antioxidant properties of triticale noodles, this study examined the feasibility of partially replacing triticale with SP powder. Due to the high protein content of SP, the fortification undoubtedly improved the protein quality of the noodles, as indicated by the increases in the protein content and in vitro digestibility. Meanwhile, because of the low eGI, the triticale noodles enriched with SP may be more beneficial for those who control their postprandial blood sugar, such as diabetics and insulin-resistant subjects. Nevertheless, demonstrating the capacity to reduce glycemic index in vivo remains a prerequisite for endorsing SP noodles as a health-promoting food. As expected, the fortification also improved the antioxidant activity of the noodles, which may be partly ascribed to the abundant polyphenolics in SP. As revealed by the color, texture, and flavor analysis, the SP addition evidently changed the sensory properties of the noodles. Generally, a 15% addition was superior in the nutritional and biological activities, but the consumer acceptability needs to be assessed for further determination.

## Figures and Tables

**Table 1:** Formulation of triticale noodles.

Sample Code	Triticale Flour (g/100 g)	BAP Powder (g/100 g)	YAP Powder (g/100 g)	BP Powder (g/100 g)
C	100	0	0	0
BAP5	95	5	0	0
BAP10	90	10	0	0
BAP15	85	15	0	0
YAP5	95	0	5	0
YAP10	90	0	10	0
YAP15	85	0	15	0
BP5	95	0	0	5
BP10	90	0	0	10
BP15	85	0	0	15

BAP, YAP, and BP represent black *Antheraea pernyi* pupae, yellow *Antheraea pernyi* pupae, and *Bombyx mori* pupae, respectively. C represents the noodles with no supplementation of insects. BAP5, BAP10, and BAP15 represent the noodles supplemented with 5%, 10%, and 15% of BAP, respectively; YAP5, YAP10, and YAP15 represent the noodles supplemented with 5%, 10%, and 15% of YAP, respectively; BP5, BP10, and BP15 represent the noodles supplemented with 5%, 10%, and 15% of BP, respectively.

**Table 2:** Electronic nose sensors and their corresponding aroma type.

Sensor Name	Aroma Type
W1C	Aromatic components
W5S	Nitrogen oxides
W3C	Aromatic and ammonia components
W6S	Hydrides
W5C	Aromatic components (short-chain alkanes)
W1S	Methyl compounds
W1W	Inorganic sulfides, terpenes
W2S	Alcohols, aldehydes, and ketones
W2W	Aromatic components (organic sulfides)
W3S	Long chain alkanes

**Table 3:** Proximate composition of triticale flour and silkworm pupa powder.

Sample	Protein (%)	Fat (%)	Ash (%)	Carbohydrate (%)
TF	19.32 ± 0.57 ^d^	1.51 ± 0.06 ^d^	2.13 ± 0.09 ^b^	77.05 ± 0.67 ^a^
BAP	55.88 ± 0.55 ^b^	25.56 ± 0.08 ^c^	2.18 ± 0.07 ^b^	16.38 ± 0.42 ^b^
YAP	57.31 ± 0.65 ^a^	28.18 ± 0.63 ^b^	1.98 ± 0.08 ^b^	12.54 ± 0.26 ^c^
BP	49.22 ± 0.93 ^c^	32.80 ± 0.70 ^a^	6.09 ± 0.68 ^a^	11.89 ± 0.90 ^c^

Different letters within the same column represent significant differences between samples (*p* < 0.05). TF: triticale flour; BAP: black *Antheraea pernyi* pupae; YAP: yellow *Antheraea pernyi* pupae; BP: *Bombyx mori* pupae.

**Table 4:** Proximate composition of triticale noodles enriched with silkworm pupa power.

Sample	Protein (%)	Fat (%)	Ash (%)	Carbohydrate (%)
C	19.69 ± 0.59 ^h^	1.60 ± 0.08 ^h^	1.33 ± 0.02 ^g^	77.38 ± 0.66 ^a^
BAP5	21.89 ± 0.02 ^fg^	1.93 ± 0.07 ^g^	1.37 ± 0.02 ^fg^	74.82 ± 0.07 ^b^
BAP10	23.60 ± 0.09 ^d^	3.15 ± 0.04 ^e^	1.43 ± 0.02 ^ef^	71.81 ± 0.10 ^e^
BAP15	25.49 ± 0.11 ^b^	4.38 ± 0.03 ^c^	1.50 ± 0.02 ^e^	68.63 ± 0.13 ^g^
YAP5	22.06 ± 0.10 ^f^	2.27 ± 0.17 ^f^	1.37 ± 0.03 ^fg^	74.30 ± 0.25 ^c^
YAP10	23.96 ± 0.13 ^d^	3.59 ± 0.03 ^d^	1.45 ± 0.06 ^e^	71.01 ± 0.13 ^f^
YAP15	25.94 ± 0.18 ^a^	4.92 ± 0.08 ^b^	1.57 ± 0.08 ^d^	67.56 ± 0.18 ^h^
BP5	21.58 ± 0.04 ^g^	3.05 ± 0.14 ^e^	1.73 ± 0.03 ^c^	73.64 ± 0.17 ^d^
BP10	22.89 ± 0.17 ^e^	4.46 ± 0.05 ^c^	1.92 ± 0.03 ^b^	70.73 ± 0.25 ^f^
BP15	24.57 ± 0.19 ^c^	6.12 ± 0.14 ^a^	2.16 ± 0.03 ^a^	67.16 ± 0.27 ^h^

Values are expressed as mean ± standard deviation (*n* = 3), and values with different lowercases in the same column are considered significant differences (*p* < 0.05). C represents the noodles with no supplementation of insects. BAP5, BAP10, and BAP15 represent the noodles supplemented with 5%, 10%, and 15% of black *Antheraea pernyi* pupae, respectively; YAP5, YAP10, and YAP15 represent the noodles supplemented with 5%, 10%, and 15% of yellow *Antheraea pernyi* pupae, respectively; BP5, BP10, and BP15 represent the noodles supplemented with 5%, 10%, and 15% of *Bombyx mori* pupae, respectively.

**Table 5:** Textural properties of noodles enriched with silkworm pupa powder.

Sample	Hardness (g)	Springiness	Cohesiveness	Chewiness	Resilience
C	689.37 ± 39.78 ^f^	0.68 ± 0.19 ^b^	0.92 ± 0.07 ^ab^	442.65 ± 167.44 ^e^	0.57 ± 0.05 ^cd^
BAP5	947.84 ± 17.81 ^e^	0.85 ± 0.03 ^a^	0.95 ± 0.01 ^a^	767.73 ± 39.62 ^cd^	0.57 ± 0.02 ^d^
BAP10	1120.01 ± 86.34 ^cde^	0.94 ± 0.04 ^a^	0.95 ± 0.01 ^a^	994.94 ± 97.21 ^bcd^	0.62 ± 0.06 ^abcd^
BAP15	1724.09 ± 272.08 ^a^	0.92 ± 0.02 ^a^	0.94 ± 0.02 ^ab^	1490.52 ± 178.40 ^a^	0.60 ± 0.03 ^bcd^
YAP5	992.57 ± 163.54 ^de^	0.89 ± 0.03 ^a^	0.96 ± 0.02 ^a^	842.16 ± 138.39 ^cd^	0.68 ± 0.01 ^a^
YAP10	1292.53 ± 80.82 ^bc^	0.91 ± 0.03 ^a^	0.96 ± 0.02 ^a^	1136.84 ± 78.10 ^b^	0.63 ± 0.02 ^abc^
YAP15	1435.64 ± 82.88 ^b^	0.93 ± 0.03 ^a^	0.95 ± 0.03 ^a^	1158.21 ± 308.27 ^b^	0.57 ± 0.01 ^d^
BP5	911.45 ± 101.08 ^e^	0.85 ± 0.03 ^a^	0.95 ± 0.01 ^ab^	735.04 ± 91.57 ^d^	0.66 ± 0.02 ^ab^
BP10	1199.89 ± 123.55 ^cd^	0.91 ± 0.02 ^a^	0.94 ± 0.03 ^ab^	1033.07 ± 120.97 ^bc^	0.60 ± 0.03 ^bcd^
BP15	1490.73 ± 106.67 ^b^	0.91 ± 0.04 ^a^	0.89 ± 0.02 ^b^	1212.22 ± 162.24 ^b^	0.62 ± 0.03 ^abcd^

Values are expressed as mean ± standard deviation (*n* = 6), and values with different lowercases in the same column are considered significant differences (*p* < 0.05). C represents the noodles with no supplementation of insects. BAP5, BAP10, and BAP15 represent the noodles supplemented with 5%, 10%, and 15% of black *Antheraea pernyi* pupae, respectively; YAP5, YAP10, and YAP15 represent the noodles supplemented with 5%, 10%, and 15% of yellow *Antheraea pernyi* pupae, respectively; BP5, BP10, and BP15 represent the noodles supplemented with 5%, 10%, and 15% of *Bombyx mori* pupae, respectively.

## Data Availability

The original contributions presented in the study are included in the article, further inquiries can be directed to the corresponding authors.
